# Differential diagnosis of illness in patients under investigation for the novel coronavirus (SARS-CoV-2), Italy, February 2020

**DOI:** 10.2807/1560-7917.ES.2020.25.8.2000170

**Published:** 2020-02-27

**Authors:** Licia Bordi, Emanuele Nicastri, Laura Scorzolini, Antonino Di Caro, Maria Rosaria Capobianchi, Concetta Castilletti, Eleonora Lalle, on behalf of INMI COVID-19 study group and Collaborating Centers

**Affiliations:** 1 National Institute for Infectious Diseases ‘Lazzaro Spallanzani’ IRCCS, Rome, Italy; 2 The participating members of INMI COVID-19 study group and Collaborating Centers are acknowledged at the end of the article

**Keywords:** differential diagnosis, SARS-CoV-2, respiratory pathogens, rapid molecular assay

## Abstract

A novel coronavirus (SARS-CoV-2) has been identified as the causative pathogen of an ongoing outbreak of respiratory disease, now named COVID-19. Most cases and sustained transmission occurred in China, but travel-associated cases have been reported in other countries, including Europe and Italy. Since the symptoms are similar to other respiratory infections, differential diagnosis in travellers arriving from countries with wide-spread COVID-19 must include other more common infections such as influenza and other respiratory tract diseases.

Following the first reports of cases of acute respiratory syndrome of unknown aetiology in Wuhan City, Hubei Province, on 31 December 2019 [1], Chinese authorities have identified a novel coronavirus, now named severe acute respiratory syndrome coronavirus 2 (SARS-CoV-2), as the causative agent [2,3]. The outbreak has spread rapidly, affecting other parts of China, and cases have been recorded on several continents (Asia, Australia, Europe and North America); further global spread is likely to occur [4]. 

The spectrum of this disease in humans, now named coronavirus disease 2019 (COVID-19) [5], is yet to be fully determined. For confirmed SARS-CoV-2 infections, reported illnesses have ranged from people with little to no symptoms to people being severely ill, having pneumonia and dying [6]. Multiple body tracts may be involved, including the respiratory, gastrointestinal, musculoskeletal and neurologic tracts. However, more common symptoms are fever (83–98%), cough (76–82%) and shortness of breath (31–55%) [6,7]. These nonspecific symptoms are shared by many other frequent infectious diseases of the respiratory tract caused by bacteria and viruses, most of which are self-limiting but may also progress to severe conditions [8,9]. Among these, the most relevant is influenza, usually characterised by fever, myalgia, headache and non-productive cough, that may also cause complications with high morbidity and mortality rate, such as pneumonia, myocarditis, central nervous system disease and death [10,11]. In addition, other previously known human coronaviruses cause similar, although milder clinical signs, including the alphacoronaviruses 229E and NL63, and the betacoronaviruses OC43 and HKU1, while two other coronaviruses, SARS-CoV and MERS-CoV, cause severe respiratory syndrome in humans [12].

The Laboratory of Virology at the National Institute for Infectious Diseases ‘Lazzaro Spallanzani’ (INMI) in Rome is the Regional Reference Centre for emerging infections and performs also diagnostics for other Italian regions without the diagnostic capability for emerging pathogens. Early in January 2020, following the announcement of the emerging outbreak, the Laboratory established the diagnostic capability for SARS-CoV-2 diagnosis and provided support to other Italian regions.

Here we focus on the results of the differential diagnosis performed on the first 126 suspected cases, analysed in the reference laboratory from 21 January to 7 February 2020.

## Diagnostic algorithm

The diagnostic algorithm adopted by the Laboratory for SARS-CoV-2 testing included, immediately upon sample receipt, a rapid molecular test for the most common respiratory pathogens in order to obtain a fast differential diagnosis. SARS-CoV-2 testing was based on the protocol released by the World Health Organization (WHO) [13], and three positive patients have been identified at the time of writing this paper. 

The 126 patients were considered suspected cases on the basis of information collected on clinical and epidemiological grounds, i.e. suspicion of a viral aetiology, recent travel history to Asia, or contact with a probable or confirmed case, according to WHO guidelines [14]. They included 64 male and 62 female cases, 52 were Italian citizens, 64 Chinese and six had other nationalities. The mean age was 35 years (range: 1–85 years).

Nasopharyngeal swab specimens were collected from 54 patients hospitalised in the high isolation facility at the INMI in Rome, nine patients from other hospitals in the Lazio Region and a further 63 cases referred to the INMI Laboratory of Virology from different regions of Italy (Figure).

**Figure:**
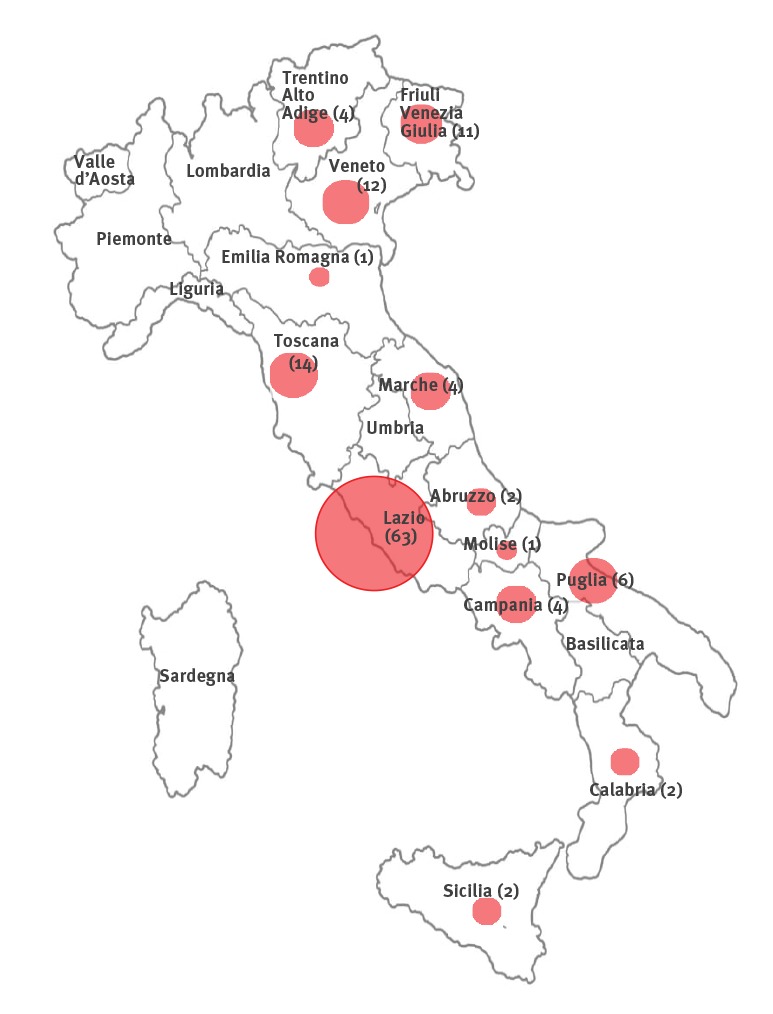
Origin (region) and number of patients tested for SARS-CoV-2 infection, Rome, Italy, 21 January–7 February 2020, (n = 126)

SARS-CoV-2: Severe acute respiratory syndrome coronavirus 2.

Samples from suspected cases tested at the Laboratory of Virology of the National Institute for Infectious Diseases ‘Lazzaro Spallanzani’. The dimension of round symbols correlates with the number of patients analysed.

## Molecular assays for respiratory pathogens

Rapid differential diagnosis was based on the QIAstat-Dx respiratory panel (QIAGEN, Milan, Italy); this system uses a single-use cartridge that includes all reagents needed for nucleic acid extraction, amplification and detection of the most common bacteria and viruses causing respiratory syndromes: adenovirus, bocavirus, coronavirus 229E (HCoV 229E), coronavirus HKU1 (HCoV HKU1), coronavirus NL63 (HCoV NL63), coronavirus OC43 (HCoV OC43), human metapneumovirus (HMPV), influenza A, influenza A subtype H1N1/pdm09 influenza A subtypes H1 and H3, influenza B, parainfluenza virus 1–2-3–4 (PIV 1–4), respiratory syncytial virus A/B (RSV A/B), rhinovirus/enterovirus, (HRV/EV), *Bordetella pertussis, Legionella pneumophila, Mycoplasma pneumoniae*. Results were available 70–90 min after sample receipt. The results considered in the analysis include those from 109 suspected cases undergoing rapid molecular testing at the Reference Laboratory, and those from 17 patients tested at the laboratory of origin (either with the rapid respiratory panel or with other methods).

Among the first 126 patients evaluated at the reference Laboratory at INMI, only three were confirmed to be infected with SARS-CoV-2 and none of those three was co-infected with other pathogens (Table).

**Table:** Viral and bacterial agents detected in patients tested for SARS-CoV-2 infection, Rome, Italy, 21 January–7 February 2020, (n = 126)

Pathogens	Patients with pathogen detected	
	n	%
Infections with a single pathogen		
None	56	44.4
SARS-CoV-2	3	2.4
Influenza A(H1N1)pdm09	12	9.5
Influenza A(H3N2)	11	8.7
Influenza A^a^	3	2.4
Influenza B	10	7.9
HRV/EV	9	7.1
HMPV	3	2.4
H-CoV 229 E	2	1.6
H-CoV NL63	2	1.6
H-CoV HKU1	2	1.6
*Mycoplasma pneumoniae*	5	4.0
*Legionella pneumophila*	1	0.8
*Streptococcus pneumoniae*	1^b^	0.8
Mixed infections		
Influenza A(H3N2) + RSV A/B	2	1.6
HRV/EV + RSV A/B	1	0.8
HRV/EV + influenza B	1	0.8
HMPV + adenovirus	1	0.8
H-CoV 229E + influenza B	1	0.8

EV: enterovirus; HMPV: human metapneumovirus; HRV: rhinovirus; SARS-CoV: Severe acute respiratory syndrome coronavirus 2.

^a^ Untyped influenza A because of low viral load.

^b^ *S. pneumoniae* was referred by the laboratory of origin.

Overall, 67 (53.2%) of the patients resulted positive for respiratory pathogen(s) other than SARS-CoV-2. Influenza viruses represented the majority of positive findings, influenza A accounting for 26 (20.6%) and influenza B accounting for 10 (7.9%) of all single infections. Other viruses were represented as well (Table). As far as bacterial infections are concerned, *M. pneumoniae* was detected in five patients (4.0%), while *L. pneumophila* and *Streptococcus pneumoniae* (the positive result for *S. pneumoniae* was referred by the laboratory origin), were found only in one patient (0.8%). Mixed infections were also observed in a small number of cases (Table).

## Discussion

Our results highlight the importance of differential diagnosis in travellers arriving from countries with widespread occurrence of COVID-19, considering the similarity of symptoms shared with more common respiratory infections, such as influenza and other respiratory tract diseases.

Broad screening for respiratory pathogens revealed a high rate of influenza virus infections, accounting for 28.5% of all suspected cases of SARS-CoV-2 infection; this is consistent with the fact that we are the middle of the seasonal influenza epidemic period. Notably, rapid influenza diagnosis facilitates timely administration of antiviral therapy, thus reducing severity and length of the disease and use of unnecessary antibiotics.

Our results highlight the importance of using a broad-spectrum molecular diagnostic panel for rapid detection of the most common respiratory pathogens, in order to improve evaluation and clinical management of patients with respiratory syndrome consistent with COVID-19. This is important in an epidemiological situation with low circulation of SARS-CoV-2, where alternative diagnoses may clarify an individual patient’s risk and may allow adjusting public health containment measures. Nevertheless, it is mandatory to maintain high level of attention with respect to this new emergent pathogen and health authorities should remain vigilant, increasing their capacity for surveillance and constantly reviewing their pandemic preparedness plans.
